# Homozygous missense variant in *BMPR1A* resulting in BMPR signaling disruption and syndromic features

**DOI:** 10.1002/mgg3.969

**Published:** 2019-09-07

**Authors:** Bianca E. Russell, Diana Rigueur, Kathryn N. Weaver, Kristen Sund, Janet S. Basil, Robert B. Hufnagel, Cynthia A. Prows, Alan Oestreich, Lihadh Al‐Gazali, Robert J Hopkin, Howard M. Saal, Karen Lyons, Andrew Dauber

**Affiliations:** ^1^ Division of Human Genetics Cincinnati Children’s Hospital and University of Cincinnati College of Medicine Department of Pediatrics Cincinnati OH USA; ^2^ Department of Molecular, Cell & Developmental Biology UCLA Los Angeles CA USA; ^3^ Department of Radiology Cincinnati Children’s Hospital Cincinnati OH USA; ^4^ Department of Pediatrics United Arab Emirates University Abu Dhabi United Arab Emirates; ^5^ Department of Orthopaedic Surgery UCLA Los Angeles CA USA; ^6^ Division of Endocrinology Cincinnati Children’s Hospital and University of Cincinnati College of Medicine Department of Pediatrics Cincinnati OH USA; ^7^ Present address: Department of Pediatrics Division of Genetics UCLA Los Angeles CA USA; ^8^ Present address: Division of Endocrinology Children's National Health System Washington DC USA

**Keywords:** atrial septal defect, BMP, bmpr1a protein, bone morphogenetic protein, human

## Abstract

**Background:**

The bone morphogenetic protein (BMP) pathway is known to play an imperative role in bone, cartilage, and cardiac tissue formation. Truncating, heterozygous variants, and deletions of one of the essential receptors in this pathway, Bone Morphogenetic Protein Receptor Type1A (*BMPR1A*), have been associated with autosomal dominant juvenile polyposis. Heterozygous deletions have also been associated with cardiac and minor skeletal anomalies. Populations with atrioventricular septal defects are enriched for rare missense *BMPR1A* variants.

**Methods:**

We report on a patient with a homozygous missense variant in *BMPR1A* causing skeletal abnormalities, growth failure a large atrial septal defect, severe subglottic stenosis, laryngomalacia, facial dysmorphisms, and developmental delays.

**Results:**

Functional analysis of this variant shows increased chondrocyte death for cells with the mutated receptor, increased phosphorylated R‐Smads1/5/8, and loss of Sox9 expression mediated by decreased phosphorylation of p38.

**Conclusion:**

This homozygous missense variant in BMPR1A appears to cause a distinct clinical phenotype.

## 1 INTRODUCTION

The bone morphogenetic protein (BMP) pathway plays an essential role in the differentiation of mesenchymal cells into chondrocytes during endochondral ossification, the process of bone formation from a cartilage template (Dunn et al., 1997; Kronenberg, 2003; Liu et al., 2005; Lowery & Rosen, 2018; Yoon & Lyons, 2004; Yoon et al., 2005). BMPs are members of the TGFβ superfamily and bind to the BMP receptors to activate the intracellular Smads 1, 5, and 8 (Aubin, Davy, & Soriano, 2004; Lowery & Rosen, 2018; Wang, Rigueur, & Lyons, 2014). The Smads then regulate transcription within the nucleus (Shi & Massagué, 2003). BMPs are also involved in the activation of *TAK1* and downstream effectors including p38 (Greenblatt, Shim, & Glimcher, 2010; Greenblatt, Shim, Zou, et al., 2010; Liu et al., 2018). Bone Morphogenetic Protein Receptor Type1A *(BMPR1A)* (OMIM: 601299) is one of the crucial membrane receptors of this pathway in skeletal tissues (Rigueur et al., 2015; Yoon et al., 2005).

Heterozygous nonsense mutations in *BMPR1A* are known to cause autosomal dominant juvenile polyposis (Cheah et al., 2009; Howe et al., 2001). Deletions of 10q22‐q23, which include *BMPR1A*, have been reported with a variable phenotype that includes cardiac septal defects, scoliosis, short stature, macrocephaly, developmental delays, and juvenile polyposis (Breckpot et al., 2012; Dahdaleh, Carr, Calva, & Howe, 2012). Heterozygous missense variants in *BMPR1A* have been reported in association with atrioventricular septal defects (D'Alessandro et al., 2016). To date, biallelic variants in *BMPR1A* have not been reported to cause human disease.

In mice, conditional knockout of *Bmpr1a* in chondrocytes results in mice with a smaller thoracic cavity, mild chondrodysplasia, and short long bones (Yoon et al., 2005). Complete loss of *Bmpr1a* in all tissues results in embryonic lethality (Mishina, Suzuki, Ueno, & Behringer, 1995). Further studies have shown that *Bmpr1a* signaling is essential for atrioventricular canal formation in early embryonic development (Kaneko et al., 2008; Park et al., 2006).

We report a patient with a homozygous missense variant in *BMPR1A* with skeletal findings, cartilaginous airway defects, cardiac anomalies, facial dysmorphisms, and developmental delays.

### 1.1 Clinical report

Our patient was a 17‐month‐old female from the United Arab Emirates born via caesarian section for fetal distress and intrauterine growth retardation at 37 weeks of age. This was the third pregnancy for her consanguineous 30‐year‐old mother and 40‐year‐old father. Her birth weight was 1.52 kg (*Z *= −3.8), length of 39 cm (*Z* = −6.8), and her head circumference was 30 cm (*Z *= −4.9). She required a 1‐month stay in the neonatal intensive care unit due to respiratory insufficiency. Her growth continued to be poor with significant developmental delays. At 8 months of age, she was hospitalized for respiratory failure. Her initial echocardiogram identified a large atrial septal defect with biatrial enlargement, normal left ventricle, size and diastolic dysfunction concerning for a restrictive cardiomyopathy. She also had a history of atrial fibrillation. Cardiac catheterization at 11 months of age confirmed the atrial septal defect, left ventricular noncompaction, and identified normal systolic function. No surgical intervention was recommended. There were severe subglottic stenosis and laryngomalacia requiring serial tracheal dilations. Given her critical airway, she was unsafe for sedation, so no brain MRI or CT imaging was obtained. She had a normal audiology evaluation, and there were no known ophthalmologic concerns.

**Figure 1:**
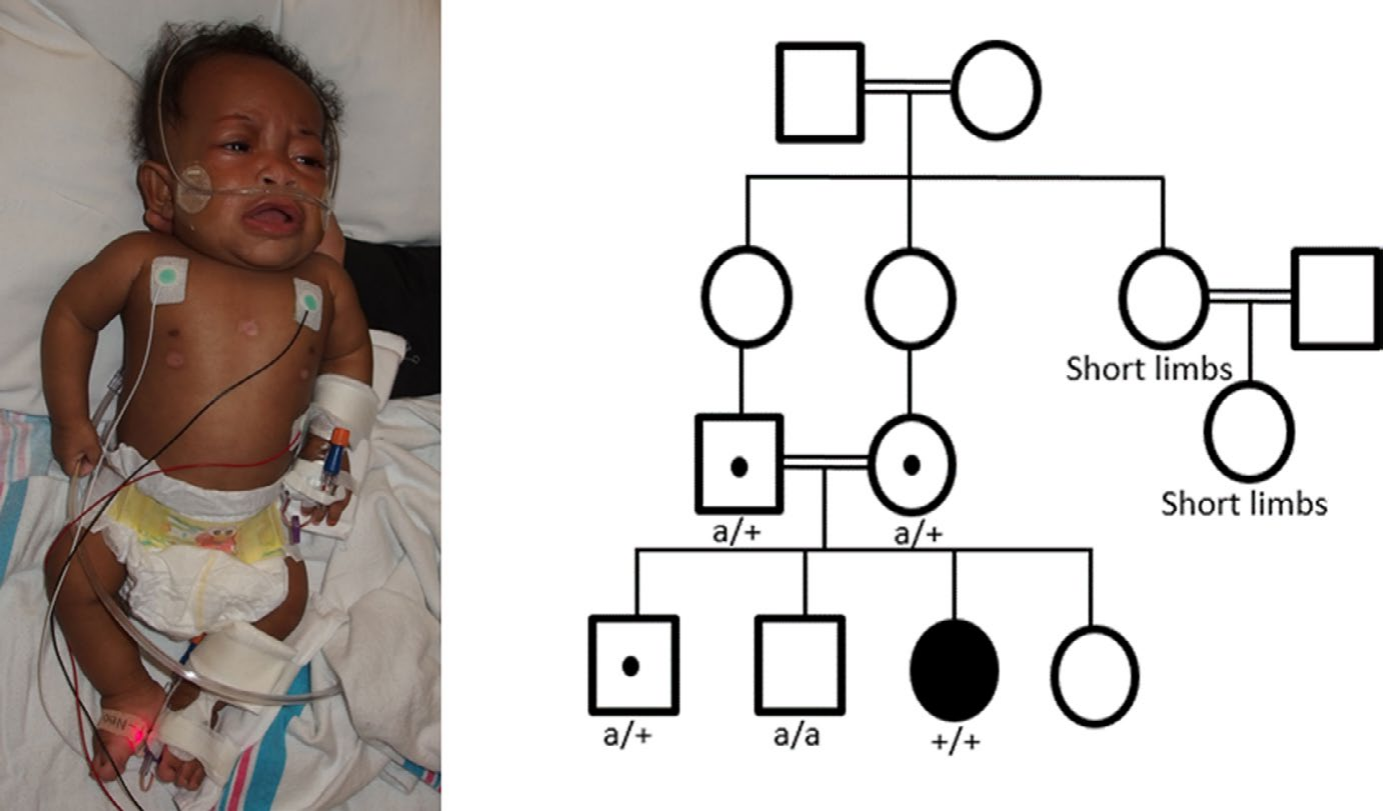
Photograph of patient with *BMPR1A* ^R406L^ homozygous variant at 10 months of age and familial pedigree. Pedigree annotated with genotypes where (+) represents *BMPR1A* ^R406L^ and (a) is the unaffected *BMPR1A* allele. All persons without a genotype are untested

On examination at 10 months of age, she had global delays with recent initiation of babbling, the ability to hold but not transfer objects and the inability to roll or sit. She did track faces and had a social smile. Her length was 47 cm (*Z* = −8), weight was 4.1 kg (*Z *= −7.3), and head circumference was 40 cm (*Z* = −3.8) with relative macrocephaly. On clinical examination, she had coarse facial features with a flat facial profile, midface retrusion, and sparse eyebrows. Her mouth was large with down turned corners and a thin upper lip. She had low set cupped ears with a short neck. She was hypotonic with poor head control and had shortened extremities with brachydactyly (Figure 1).

**Figure 2:**
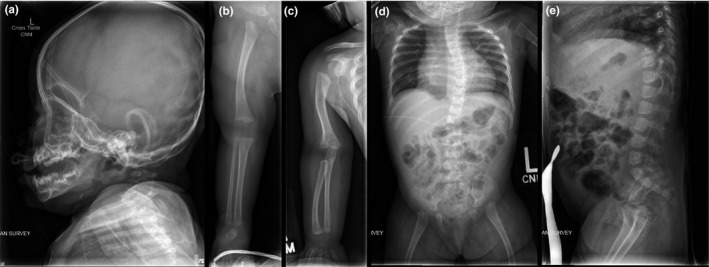
Selected skeletal images at 10 months of age. (a) Brachycephaly associated with subtle unilateral coronal synostosis (not seen in this image). (b) Lateral bowing of right femur, proximal tibia growth center not yet ossified. (c) Lateral bowing of right humerus and radius. (d) Mild cardiomegaly and mild thoracic scoliosis. Subluxation of the right hip and luxation of the left hip with no ossification of the capital femoral epiphysis. (e) Some posterior wedging of L1 and L2 bodies

X‐rays demonstrated mild osteopenia from reduced muscular activity. She had a discordant bone age of not more than 3 months (biological age 10 months) including small secondary growth centers. She had notable brachycephaly with subtle unilateral coronal synostosis. Scoliosis was present with a 34° curvature and posterior wedging of vertebral bodies L1 and L2. Her hips were dysplastic with poorly formed, bilateral dysplastic acetabula. She exhibited subluxation of the right hip and luxation on the left with no ossification of the capital femoral epiphysis. The long bones laterally demonstrated convex bowing that was more significant in the proximal portion of the extremities. She had mild brachydactyly (Figure 2).

Her family history was significant for consanguinity as her parents were first cousins. She had three healthy siblings and her mother did not have any pregnancy losses. Family members did not have cardiac imaging. There were two distant relatives with short stature, short limbs, and possibly similar facial features who were adults without any known cardiac anomalies (Figure 1).

Initial testing included a SNP microarray with 9.97% homozygosity, negative *FGFR3* sequencing and normal lysosomal storage enzyme levels. She also had normal CPK levels, urine organic acids, and plasma amino acids.

At 17 months of age, she died due to presumed aspiration pneumonia in her home country. No autopsy was performed.

## 2 METHODS

### 2.1 Editorial Policies and Ethical Considerations

Parental consent for research and photo publication was attained prior to sample collection.

### 2.2 Sequencing

DNA samples were collected from the proband, parents, and two healthy brothers. A research‐based exome was completed by the Cincinnati Children's DNA Sequencing and Genotyping Core using Illumina's Hi Seq 2500. The Broad Institute's web‐based Genome Analysis Toolkit was used. Variants were analyzed within Genome Reference Consortium Build 37, hg19. Sanger sequencing was done to confirm only the primary variants of interest, *USH2A* and *BMPR1A*.

### 2.3 Functional studies

#### 2.3.1 Primary Chondrocyte Isolation

Isolation of primary costal chondrocytes was conducted as stated in Lefebvre et al., 1994 and Mirando, Dong, Kim, & Hilton, 2014. In brief, primary costal chondrocytes were isolated from neonatal 0‐day old mice (P0). Rib cages were isolated, rinsed twice in 1x PBS, and incubated in Pronase solution (2mg/ml [Sigma‐Aldrich; cat# 10165921001] in DMEM) for 30 min at 37°C on a rocker. Cells were then washed and shaken by hand thoroughly three times with 1x PBS until intercostal muscle and surrounding tissue were visibly removed from rib cage chondrocytes. The solution was aspirated between washes. Clean ribcages were then placed in a 3mg/ml of Collagenase Type II solution dissolved in DMEM and 1% Pen/Strep (Collagenase Type II [Gibco 17101‐05]). This solution was filter‐sterilized before use. Rib cages were incubated for 1.5 hr at 37°C on a rocker and checked for the level of single cell suspension. Cells were incubated for another hour on the rocker at 37°C. Cells were then spun down at 1000 rounds per minute for 5 min. Cells were washed 3x in 1x PBS and then re‐suspended in chondrogenic medium (DMEM supplemented with 10% FBS, 1% Pen/Strep Glutamine [Gibco 10378‐016] and 50mg/ml ascorbic acid). Cells were seeded at 1 × 10^5^ cells/well in 12‐well plates and allowed to recover overnight. The next day the media were replaced. This experiment was performed independently three times (i.e., from three to four different cell isolations).

#### 2.3.2 Site‐directed mutagenesis and transfections

Using one step PCR site‐directed mutagenesis, a construct encoding the human BMPR1A^R406L^ variant was generated in pcDNA3‐ALK3/BMPR1A (Addgene). Mouse chondrocytes were isolated from neonatal mice and seeded at 1 x10^5^ in 12‐well plates and allowed to recover for 24 hr. Cells were subsequently serum starved for 4 hr, transfected with lipofectamine 2000 and either 300 ng of empty vector, wild‐type *BMPR1A/ALK3,* or *BMPR1A/ALK3^R406L^* (pcDNA3‐eGFP, pcDNA3‐ALK3^WT^, pcDNA‐ALK3^R406L^). The cells grew for 3 days and then cell viability was assessed using a trypan blue exclusion assay. This process was repeated in triplicate (i.e., from three different cell isolations).

#### 2.3.3 Western blot analysis

Mouse chondrocytes were isolated from neonatal mice and transfected as stated above (300ng of pcDNA3‐eGFP, pcDNA3‐ALK3^WT^, or pcDNA‐ALK3^R406L^). Thirty‐two hours post transfection, cell lysates were serum starved for 4 hr and treated with or without 50 ng of BMP‐2 ligand in DMSO (R&D Systems, 355‐BM), or with an equal volume of DMSO for untreated cells. Lysates were generated using EBC lysis buffer (50 mM Tris, 120 mM NaCl, 0.5% Nonidet P‐40), supplemented with a protease inhibitor mixture (Complete Mini Tablets, Roche Applied Science, Indianapolis, IN, USA) and phosphatase inhibitors (Sigma‐Aldrich, P5726) on ice (Rigueur et al., 2015). Whole cell lysates (20–30 µg) were loaded in 10% polyacrylamide gels and were subjected to SDS‐PAGE. Proteins were then transferred onto PVDF membranes. The membranes were blocked for 1 hr in 5% milk or 5% BSA in TBS‐tween 20 (30 mM Tris pH 7.4, 300 mM NaCl, 0.2% tween‐20), incubated with primary antibody (from Cell Signaling Technology: Sox9 [#82630], p38 [#9212], phospho‐p38 (p‐p38) [#9211], phospho‐Smad1/5/8 (p‐Smad1/5/8) [#9511]), ß‐actin [#4967]; from Sigma‐Aldrich: tubulin [#T6793]; from Abcam: BMPR1A [#ab38560]; and from ThermoFisher Scientific: BMPR1A [#38‐6000]) were diluted in blocking buffer overnight at 4°C, and then incubated with appropriate secondary antibody diluted in blocking buffer for 1 hr at room temperature. Antibody binding was detected via enhanced chemiluminescence using the Pierce ECL plus Western blotting Substrate (ThermoFisher Scientific [#32132]).

#### 2.3.4 RNA Isolation and Quantitative Real‐Time RT‐PCR

RNA isolation was conducted per RNeasy Kit (Qiagen) manufacturers' instructions. Synthesis of cDNA was performed employing Superscript III (Invitrogen). Quantitative real‐time PCR was performed using SYBR Green Real‐Time PCR Master Mix (Fermentas) on an MX3005P QPCR System (Stratagene). Primer sequences were as follows: ß‐actin forward 5’‐CTGAACCCTAAGGCCAACCG‐3”, reverse 5’‐GTCACGCACGATTTCCCTCTC‐3’; Sox 9 forward 5’‐AGGCCACGGAACAGACTCA‐3’, reverse 5’‐ AGCTTGCACGTCGGTTTTG‐3’.

## 3 RESULTS

### 3.1 Sequencing

Whole exome sequencing of the proband, parents, and two healthy brothers identified 96,137 variants with 31,342 variants being nonsynonymous. Variants were filtered for a minor allele frequency of <0.001 and familial segregation. The variants were analyzed by multiple members of the research team and 14 variants of interest that fit the above criteria were discussed with an expert panel of researchers and clinical geneticists at Cincinnati Children's Hospital. All 14 variants with protein predictions are listed in Table S1. Of note, there was an incidental variant in *USH2A*. Of the variants of interest, the only variant associated with possible skeletal, cardiac, and cartilage defects was a homozygous missense variant in *BMPR1A*.

**Figure 3:**
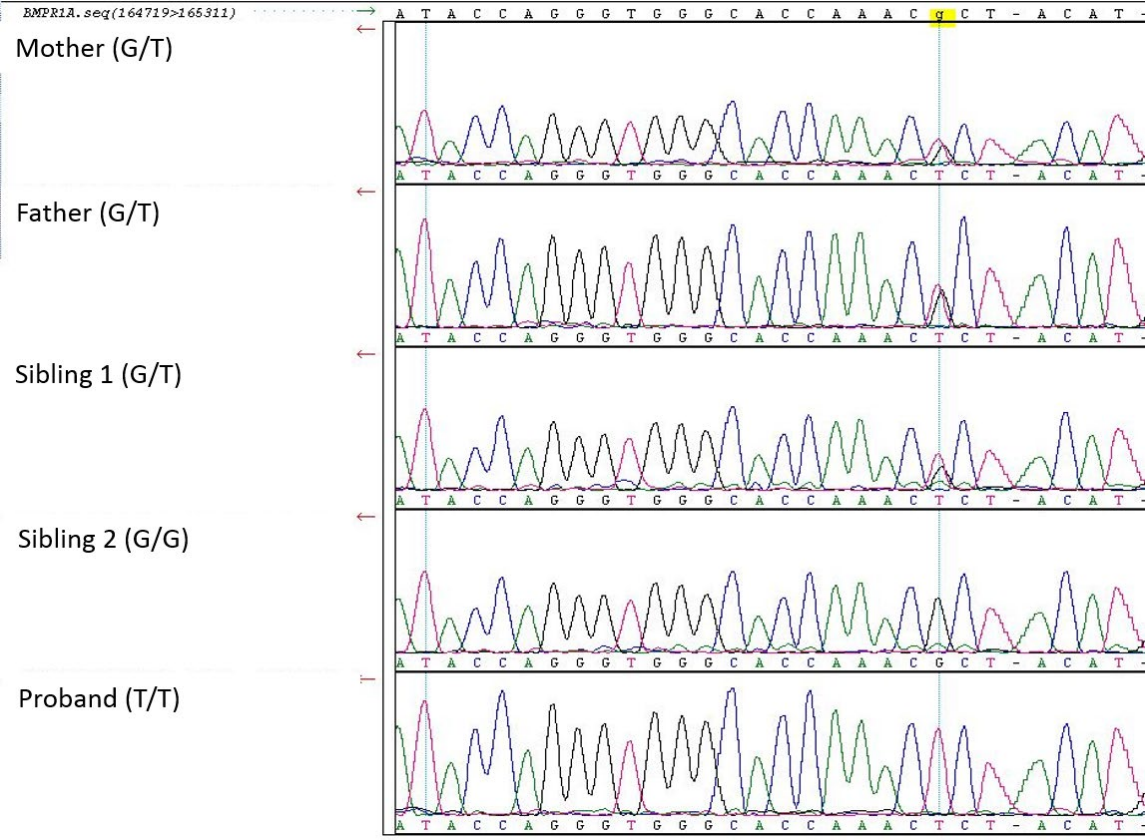
Sanger sequencing results for *BMPR1A* c.1217G > T p.R406L. Genotype of each family member depicted in parenthesis

The proband possessed two copies of a variant in *BMPR1A* NM_004329.2 (10q23.2 c.1217G > T p.Arg406Leu) which was inherited from both heterozygous parents and is located in a region of homozygosity identified on SNP microarray. One brother was heterozygous for this variant while the other did not carry the variant. This variant was Sanger confirmed (Figure 3). This variant in *BMPR1A* is predicted to be damaging in five out of six protein prediction software programs (Mutation Taster, SIFT, PPH2, Mutation Assessor, FATHMM, and FATHMM MLK Coding). The variant is located in the encoding region of the protein kinase domain of BMPR1A, and it changes the terminal amino acid in an activation loop (A‐loop) from an arginine to a lysine. It is also a novel variant in a highly conserved residue down to *C. elegans*, which has not been reported in gnomAD, ExAC, Greater Middle East Variomes, HGVD, or ClinVar. Similar variants that result in p. Arg406Cys (10–88681326‐C‐T) and Arg406His (10‐88681327‐G‐A) are reported in gnomAD with an allele frequency of 7.78 × 10^−5^ and 3.98 × 10^−6^. These variants are in the Finnish and broader European population and they have not been reported to exist in the homozygous state. This variant was uploaded to ClinVar https://www.ncbi.nlm.nih.gov/clinvar/.

### 3.2 Functional Studies in Primary Mouse Chondrocytes

**Figure 4:**
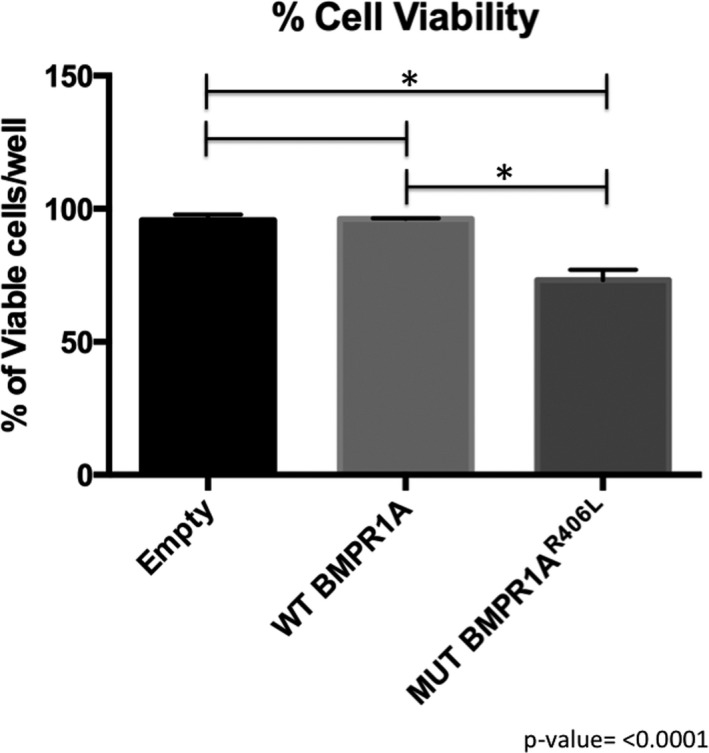
Cell viability in chondrocytes. Cells transfected with empty vector, *BMPR1A^WT^*, or *BMPR1A^R406L^* were analyzed for cell survival (*p < .0001)

**Figure 5:**
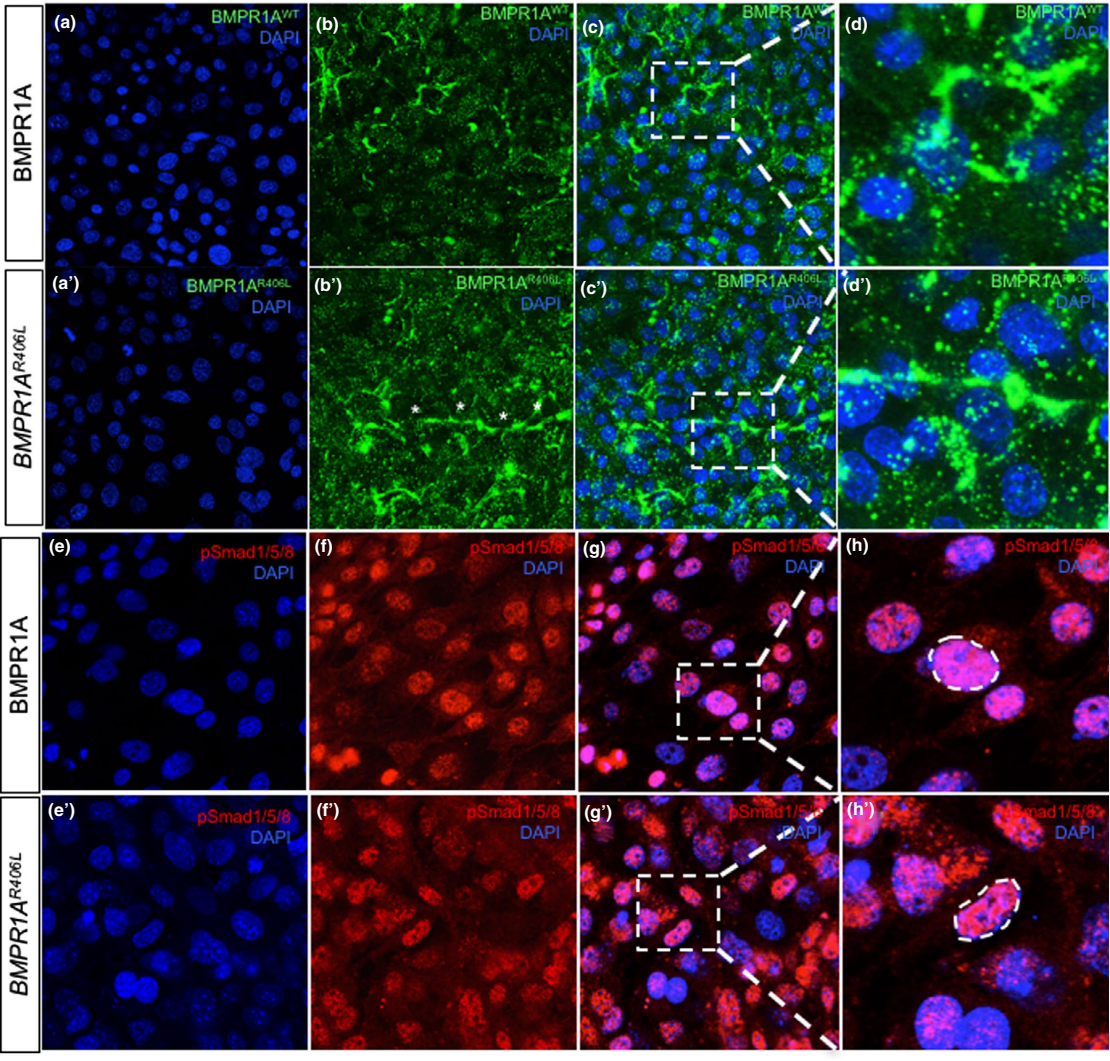
BMPR1A and phosphor‐Smad1/5/8 localization in transfected chondrocytes. (a, a’) Nuclear staining with DAPI of chondrocytes transfected with *BMPR1A^WT^* on the top left panel, or *BMPR1A^R406L^* in the panel below, are shown in blue at 20x. (b, b’) Immunofluorescence of the proteins BMPR1A^WT^ or BMPR1A^R406L^ were conducted using a His‐tag antibody, shown in green. Cells display receptor localization limited to the cell surface (c, c’). Merged fluorescent images of cells stained for DAPI and products of the transfected *BMPR1A^WT/R406L^* constructs highlight the receptor's localization to the cell surface. A square was drawn around the areas that were magnified and shown in the adjacent right panels. (d, d’) Images of transfected cells probed for BMPR1A^WT^ and BMPR1A^R406L^ are magnified at 63x. (e–h) Images of cells transfected, like the panels above, were probed for DAPI, pSmad1/5/8, merged at 40x, and magnified at 63x for resolution of pSmad1/5/8 cytoplasmic and nuclear localization in BMPR1A^WT^ and BMPR1A^R406L^ expressing cells (e’–h’)

**Figure 6:**
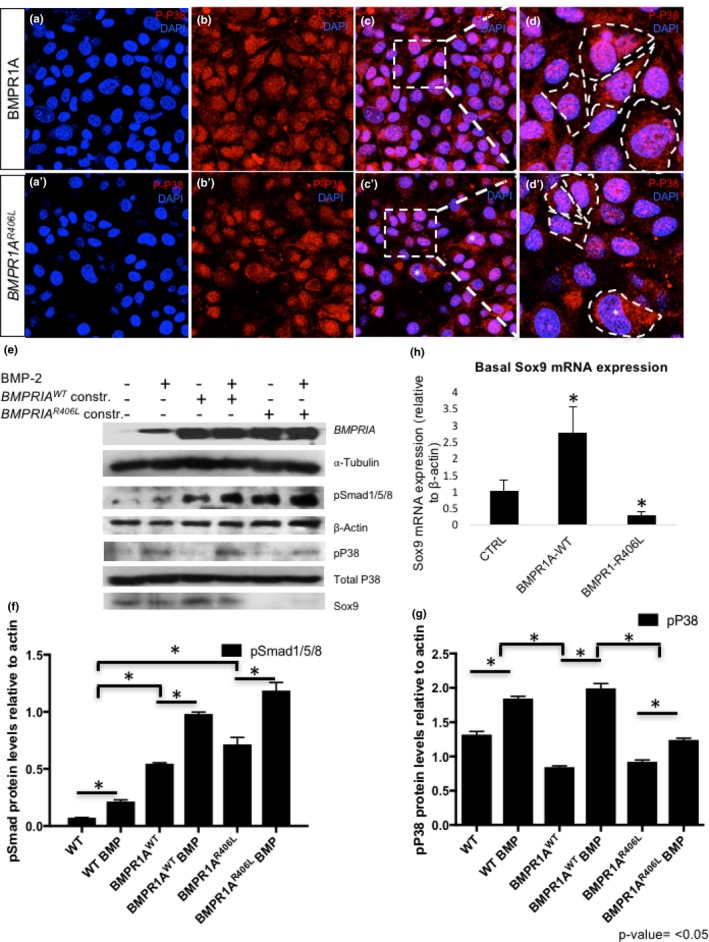
Reduced p‐p38 and loss of Sox9 expression in chondrocytes transfected with *BMPR1A R406L* variant. (a, a’) Nuclear staining with DAPI of chondrocytes transfected with *BMPR1A^WT^* on the top left panel, or *BMPR1A^R406L^* in the left panel below, are shown in blue at 20x. (b, b’) Immunofluorescence of p‐p38, shown in red, displays both cytoplasmic and nuclear localization in BMPR1A^WT^ and in BMPR1A^R406L^‐expressing cells; however, a reduction is shown in b’. (c, c’) Merged fluorescent images of cells probed for DAPI and p‐p38 show a subset of cells with p‐p38 nuclear localization. A square is drawn around the areas that were magnified and shown in the adjacent right panels and a star (*) designates cells that have reduced nuclear p‐p38. (d, d’) Images of transfected cells probed for p‐p38 are magnified at 63x. White‐dashed lines highlight the shapes of the cells. (e) Western blot analysis of BMPR1A, loading control alpha‐tubulin, phospho‐Smad1/5/8 (pSmad1/5/8), loading control beta‐actin, phospho‐ and total p38, and Sox9 were conducted. (f, g) Western blot protein levels of p‐Smad1/5/8 and p‐p38, respectively, were quantified and graphed relative to loading control beta‐actin. (h) Basal levels of Sox9 mRNA expression were detected and quantified by qRT‐PCR in cells transfected with vector control, *BMPR1A^WT^*, or *BMPR1A^R406L^* (*p‐value < .05)

Mouse chondrocytes transfected with the human *BMPR1A^R406L^* construct demonstrated chondrocyte cell death associated with overexpression of the mutated form of BMPR1A*.* Approximately 30% of cell loss was observed after 3 days of growth as compared to empty vector controls or cells transfected with *BMPR1A^WT^* expression vector (*p* < .05) (Figure 4). Transfection efficiency and localization of the wild‐type and mutant receptor were visualized through immunofluorescence and via western blot analysis (Figures 5 and 6, respectively). Both the wild‐type and mutated receptor were localized predominantly on the cell surface (Figure 5a–d; a'–d'). Immunostaining for phospho‐Smad1/5/8 (p‐Smad1/5/8) revealed no obvious differences in basal activation of canonical BMP pathways in cells overexpressing BMPR1A^WT^ compared to BMPRIA^R406L^, through fluorescence (Figure 5e–h; e’–h’). We next examined activation of noncanonical pathways by immunofluorescence for phospho‐p38 (p‐p38) (Ionescu et al., 2003). No obvious visible differences were seen (Figure 6a–d; a’–d’). Overall, cells expressing the mutant receptor exhibited reduced viability, but the remaining cells appeared to engage in BMP pathway activity.

### 3.3 Signal pathway effect

In order to test the impact of BMPR1A^R406L^ in greater detail, expression levels of molecular markers of chondrocytes and BMP signaling pathway effectors were examined. Western blot analysis was employed to quantify Sox9, p38, p‐p38, and p‐Smad1/5/8 in chondrocytes. The canonical pathway was first analyzed. Cells overexpressing the BMPR1A^WT^ or BMPR1A^R406L^ receptor, without BMP‐2 ligand stimulation, showed increased basal p‐Smad 1/5/8 levels, when compared to cells transfected with the empty vector, which had little to no levels of basal phosphorylated Smads (Figure 6e). All cells, including those overexpressing BMPR1A^WT^ or BMPR1A^R406L^, were responsive to BMP‐2 stimulation, showing increased phosphorylation of Smads1/5/8 after treatment when compared to their respective basal levels. Interestingly, the levels of p‐Smad1/5/8 were greater in cells expressing BMPR1A^R406L^ pre‐ and post‐BMP‐2 treatment when compared to cells overexpressing BMPR1A^WT^ (Figure 6e, f). This data suggests BMPR1A^R406L^ exhibits significant enhancement of canonical R‐Smad activation.

However, when the noncanonical pathway effector p38 was analyzed, the phosphorylation levels between cells overexpressing BMPR1A^WT^ or BMPR1A^R406L^ were differentially affected than that of the canonical pathway (Figure 6f). BMPR1A^R406L^‐expressing cells showed reduced ability to phosphorylate p38 pre‐ and post‐BMP‐2 treatment to comparable levels to that of cells overexpressing BMPR1A^WT^ (Figure 6e, g). In fact, the levels of p‐p38 were more closely comparable to cells transfected with the empty vector. Overall, BMPR1A^R406L^ shows enhanced ability to phosphorylate R‐Smads 1/5/8 than the wild‐type BMPR1A receptor; however, BMPR1A^R406L^ exhibits some impairment in the activation of noncanonical pathways observed through reduced levels of p‐p38 (Figure 6e, g).

The transcription factor Sox9 is a master regulator of chondrocyte lineage commitment, differentiation, and maintenance (Liu & Lefebvre, 2015). Sox9 is a direct target of BMP signaling and is regulated by both canonical and noncanonical BMP pathways in cartilage (Pan et al., 2008; Retting, Song, Yoon, & Lyons, 2009). Although enhanced Sox9 activity may result from enhanced levels of pSmad1/5/8, Sox9 expression can be induced directly by BMP‐mediated activation of p38. Phosphorylated p38 binds to the cis proximal promoter of Sox9, inducing Sox9 mRNA and protein expression. Furthermore, moderate pharmacological impairment of p38 phosphorylation has been shown to also downregulate Sox9 expression (Pan et al., 2008). We therefore examined Sox9 levels, and observed a significant reduction in cells expressing BMPR1A^R406L^ at the mRNA and protein level on the third day post‐transfection (Figure 6e, h). The addition of BMP‐2 induced some Sox9 expression in BMPR1A^R406L^‐expressing cells, but levels remained considerably lower than in cells overexpressing BMPR1A^WT^, or those transfected with empty vector (Figure 6e). This loss of Sox9 expression is consistent with the reduction of p‐p38 (Pan et al., 2008). All together, the data shows that along with reduced viability, BMPR1A^R406L^‐expressing cells have difficulty maintaining Sox‐9 expression.

In summary, overexpression of BMPR1A^R406L^ in chondrocytes resulted in enhanced p‐Smad1/5/8 levels to that of the wild‐type receptor, but impaired noncanonical BMP signaling through p38, leading to reduced cell viability and loss of Sox9 expression.

## 4 DISCUSSION

The bone morphogenetic protein (BMP) pathway has been shown in mice, as well as postulated in humans, to play an essential role in bone, cartilage, and cardiac formation. Since Bone Morphogenetic Protein Receptor Type1A *(BMPR1A)* is one of the crucial receptors in this pathway, it is logical that variants in *BMPR1A* may cause defects in the same tissue types as we report in our patient.

While variants in *BMPR1A* have been linked to human disease, the type and location of the variant seems to produce different phenotypes. Our patient has a homozygous missense variant in the protein kinase domain that has not previously been reported. The association of nonsense *BMPR1A* variants and autosomal dominant juvenile polyposis has a different inheritance pattern, and likely a separate mechanism of action (Howe et al., 2001). Patients with deletions of chromosome 10q23.2 have a range of features that may include cardiac septal defects, scoliosis, short stature, macrocephaly, developmental delays, and juvenile polyposis (Breckpot et al., 2012; Dahdaleh et al., 2012). There appears to be some phenotypic overlap between the autosomal dominant nonsense variants that cause polyposis and complete deletions of *BMPR1A* who also have polyposis. Patients with deletions also seem to possess skeletal features that may overlap with those seen in our patient but are milder. Because these deletions often include more than just *BMPR1A*, it is hard to determine if the overlapping features are caused by loss of another gene.

Enrichment of heterozygous rare or novel *BMPR1A* missense variants in a population of patients with atrioventricular septal defects (3 out of 81 patients) provides further evidence of the role of *BMPR1A* in cardiac formation (D'Alessandro et al., 2016). Interestingly, one of these patients also had learning difficulties, psychiatric concerns, and cervical spine anomalies. These three variants were all missense variants located in the protein kinase domain, similar to our patient. In the heterozygous state, it is possible that missense variants cause enough dysfunction in the BMP pathway to produce primarily cardiac defects. Homozygous missense *BMPR1A* variants could be postulated to cause a more severe phenotype involving bone, cardiac, and cartilaginous tissues as is reported in our patient.

For our patient, the clinical history fits well with what may be plausible for a homozygous missense variant given the skeletal changes, severe subglottic stenosis and laryngomalacia, facial dysmorphisms, as well as a large atrial septal defect. Given the decrease function of this variant, it is plausible that juvenile polyposis would be noted in this family. Parents have not been screened but report no family history of polyps.

Our functional studies further support that this variant disrupts BMP signaling pathways. It is conceivable that elevated canonical signaling could account for the reduced viability of BMPR1A^R406L^‐expressing cells, as enhanced BMP signaling causes apoptosis in many tissues, ranging from interdigital cell death during development (Breckpot et al., 2012; Zou & Niswander, 1996) to the craniofacial complex with aberrant conditional knockin mutations in mouse models (Hayano, Komatsu, Pan, & Mishina, 2015). Constitutively active Bmpr1a (ca‐Bmpr1a) in cranial neural crest cells results in craniofacial deformities, disrupts nasal cartilage morphogenesis, and causes apoptosis of calvarial tissues (Hayano et al., 2015; Komatsu et al., 2013; Zou & Niswander, 1996). While Smad‐dependent pathways were elevated in these studies, p38 pathways were not enhanced in the presence of constitutively active receptor, similar to our findings (Komatsu et al., 2013). Nonetheless, apoptosis ensued from only the Smad‐dependent branch of BMP signaling. Moreover, although slight, yet significant, increases in Smad1/5/8 phosphorylation were observed in BMPR1A^R406L^‐expressing chondrocytes, to that of cells overexpressing the wild‐type receptor, slight increases in BMP signaling are sufficient to cause craniofacial abnormalities. Craniofacial defects obtained in mice expressing ca‐Bmpr1a can be rescued by removal of one endogenous Bmpr1a allele (Hayano et al., 2015). Overall, these results underscore the importance of balancing BMP Smad‐dependent pathways during development.

Deficient activation of the p38 pathway observed in BMPR1A^R406L^‐expressing chondrocytes results in loss of Sox9 expression; however, it may contribute to the apoptosis phenotype observed in our cell culture. Mouse studies have shown that compound mutations in constitutively active Bmpr1a and loss of function of TAK1 (upstream of p38 activation) exacerbate deformities via increased cell death (Liu et al., 2018). Moreover, loss of noncanonical signaling via TAK1 in developing nasal cartilage results in enhanced p‐Smad1/5/8 levels, a phenotype similar to our cell transfection study (Liu et al., 2018). Although the presence of TAK1 activation in some organ systems have been shown to enhance and allow for full activation of p‐Smad1/5/8 in long bone cartilage (Gunnell et al., 2010), it appears that activation of TAK1 in cartilage from the craniofacial complex competes with R‐Smads for BMP receptor phosphorylation. Although costal chondrocytes were used in our study, their signaling phenotype more closely resembles Bmpr1a gain of function studies conducted on cartilage of the cranial axial skeleton. Overall, in our system, both elevated p‐Smad1/5/8 and reduced p‐p38 levels could contribute to the reduced viability of BMPR1A^R406L^‐expressing costal chondrocytes.

A limitation of the functional studies is that they employed overexpression of receptors. This overexpression approach may compensate, and thus mask, some of the differences in the signaling properties of BMPR1A^WT^ versus BMPR1A^R406L^. Nonetheless, the overexpression approach revealed clear differences in cell viability and maintenance of the chondrocyte maker Sox9, associated with altered BMP pathway activity.

While further identification of other individuals is needed to better define this syndrome, we suggest that the homozygous missense variant R406L in *BMPR1A* causes a recognizable pattern of clinical features in our patient.

## CONFLICT OF INTEREST

All authors have stated that they do not have any conflict of interest or disclosures.
