# Supplementary material for: Homozygous missense variant in BMPR1A resulting in BMPR signaling disruption and syndromic features
Source: Mol Genet Genomic Med. 2019 Sep 7;7(11):e969. doi: 10.1002/mgg3.969 (PMC6825850; doi:10.1002/mgg3.969)
Supplement: Supplementary file 1 [file MGG3-7-e969-s001.pdf]

Gene	Variants	Inheritance	Disease/Gene Function	gnomAD	Damaging Prediction
**BMPR1A**	c.1217G>T<br>p.R406L	Homozygous	AD juvenile polyposis, skeletal dysplasia-mice	Absent	5 of 6
**C12orf50**	c.758C>T<br>p.S248P	Homozygous	Unknown	Absent	1 of 6
**CYB561D1**	c.647T>A<br>p.I216N	Homozygous	Unknown	$7.6x10^{-4}$<br>1 homozygote	4 of 6
**EED**	c.1328T>A<br>p.F443Y	De Novo	Ectoderm development, overgrowth syndrome, Weaver-like	Absent	5 of 6
**GRID1**	c.2821C>T<br>p.R941W	Homozygous	Glutamate receptor, mice behavioral problems	$1.19x10^{-5}$<br>No homozygotes	4 of 6
**HEG1**	c.3232T>G<br>p.F1078V<br><br>c.2159C>G<br>p. P720G	Compound Heterozygous	Animal models with enlarged heart, gastrulation defects	$1.24x10^{-4}$<br>$4.87x10^{-5}$<br>No homozygotes	2 of 6<br>2 of 6
**MCOLN2**	c.760A>T<br>p.N254Y	Homozygous	Lysosome cation channel	$1.28x10^{-4}$<br>No homozygotes	6 of 6
**NR2C1**	c.132C>A<br>p.F44L	Homozygous	Steroid receptor in human testis	$3.98x10^{-6}$<br>No homozygotes	5 of 6
**NTN4**	c.1324G>A<br>p.G442R	Homozygous	Regulates cell growth in neuron/vessels	$4.1x10^{-4}$<br>No homozygotes	5 of 6
**NUP107**	c.503G>A<br>p.S168N	Homozygous	Nephrotic syndrome II, Galloway-Mowat Syndrome 7, Ovarian dysgenesis 6	$3.91x10^{-5}$<br>No homozygotes	0 of 6
**PARG**	c.2045A>G<br>p.Y682C	Homozygous	Glycohydrolase, neurodegeneration in flies	Absent	3 of 6
**SHPRH**	c.1100T>G<br>p.L367R	Homozygous	Possibly involved in DNA repair, chromatin processing	$3.25x10^{-4}$<br>No homozygotes	4 of 6
**SLC25A24**	c.62C>T<br>p.P21L	Homozygous	Fontaine progeroid syndrome	$7.11x10^{-5}$<br>No homozygotes	0 of 6
**USH2A**	c.11155C>T<br>p.R3719C	Homozygous	Usher Syndrome	$1.19x10^{-5}$<br>No homozygotes	5 of 6
